# Effect of Multimedia Education on Nutritional Behaviour for Colorectal Cancer Prevention: An Application of Health Belief Model

**DOI:** 10.21315/mjms2018.25.6.11

**Published:** 2018-12-28

**Authors:** Tooba Hatami, Azita Noroozi, Rahim Tahmasebi, Alireza Rahbar

**Affiliations:** 1 Department of Health Education & Promotion, Bushehr University of Medical Sciences, Bushehr, Iran; 2 The Persian Gulf Tropical Medicine Research Centre, The Persian Gulf Marine Biomedical Sciences Research Institute, Bushehr University of Medical Sciences, Bushehr, Iran; 3 Department of Biostatistics, Bushehr University of Medical Sciences, Bushehr, Iran; 4 Department of Nutrition, Bushehr University of Medical Sciences, Bushehr, Iran

**Keywords:** colorectal cancer prevention, electronic education, health belief model, nutritional behaviour

## Abstract

**Background:**

Colorectal cancer is a major public health problem. A way to decrease this cancer is through dietary behavioural changes. The aim of this study was to determine the effects of education on dietary behaviour based on the health belief model (HBM) using multimedia.

**Methods:**

In this clinical trial study, 98 participants were randomly allocated to an HBM group (*n* = 48) and a control group (*n* = 50). The HBM group received an audiovisual compact disc (CD) that contained information about nutritional behaviour of colorectal cancer (CRC) prevention based on HBM that lasted 45 min. Both groups completed questionnaires regarding demographic factors, knowledge and HBM constructs, and a three-day dietary recall at the beginning of the study, 1 week after, and 3 months after the education. The outcome of this study was measured by the amount of food servings consumed and dietary micronutrient intake.

**Results:**

At the baseline, there were no significant differences between groups regarding demographic factors. Findings showed that self-efficacy (*P* < 0.001), severity (*P* < 0.001), and benefits (*P* < 0.001) were perceived to be higher, and knowledge (*P* < 0.001) was increased in the HBM group compared to control group 3 months after education. There was a significant increase in fruit and vegetable (*P* < 0.001) and dairy (*P* = 0.001) intake and a significant decrease in red meat servings (*P* = 0.016) in the HBM group compared to the control group. Also, intake of vitamin D (*P* < 0.001), folate (*P* < 0.001), calcium (*P* = 0.008), and dietary fibre (*P* < 0.001) was increased in the HBM group compared to the control group 3 months after education.

**Conclusion:**

Education plans based on HBM and implemented through multimedia can change nutritional beliefs and behaviours for the prevention of colorectal cancer.

## Introduction

About one million new cases of colorectal cancer (CRC) are detected every year worldwide (1). Recent studies have shown a rapid rise in the incidence of colorectal cancer in Iran, and a meta-analysis study conducted in 2015 reported an age-standardised incidence rate of 8.16 and 6.17 (per 100,000) for Iranian men and women, respectively (2). Also, in Iran, about 15%–35% of cases occur in individuals under 40 years of age (3). This cancer was the third-most costly cancer in European countries, and according to Vahdatimanesh’s study results, the economic burden of CRC in Iran was $298 million in 2012 (4). Therefore, the increasing incidence and high economic burden of CRC in the past three decades in Iran has made it a major public health problem (3).

Today, it is estimated that 30%–40% of CRC cases are linked to nutrition and other lifestyle factors (5). The incidence of the cancer in recent years has also increased in Iran because of changes to lifestyle and diet (6). Many studies have attributed the increased risk of CRC to the prevalence of a Western diet that is high-meat, high-calorie, fat-rich, and fibre-deficient (7, 8). Studies have shown that per capita consumption of fat has increased in Iran (9, 10). Also, fast foods—another staple of the Western diet—have become increasingly prevalent over the past few years in Iran (11). Regular consumption of fish, fibre, vitamin D, folate, and calcium can prevent the development of CRC (12–15).

Dietary interventions are difficult to develop and evaluate because they frequently require changes in complex behaviours (5). Behavioural theory has increasingly been used to guide nutrition research to improve intervention efficacy (16). Several studies have indicated that dietary compliance is related to the individual’s perceptions and beliefs (5). One of the predictive models of nutritional behaviour, based on individual perceptions, is the health belief model (HBM) (17–19).

Based on this model, if people believe that they are susceptible to diseases, such as CRC (perceived susceptibility); understand the risk depth and severity of CRC complications in their life (perceived severity); consider proposed ways, including healthy eating, that can decrease risk or severity of CRC (perceived benefits); and are able to overcome obstacles for action, including cost and difficulty of healthy eating (perceived barriers), they will be more likely to participate in health-improvement programs (20).

In several studies, the effectiveness of verbal education on the preventive nutritional behaviour of osteoporosis, based on HBM, has been confirmed (21–23), as well as nutrition management in diabetic patients (24). In addition, one study has shown that verbal education based on HBM is effective for decreasing fat, saturated fat, and cholesterol intake for preventing CRC (16).

Nowadays, researchers have utilised technological developments to provide interventions for health promotion. Multimedia allows for users to easily interact with content due to its dynamic and attractive graphical effects and use of various visual and audio media. Through this method, the learner will find an opportunity to practice and reach a proficiency level. So, given the increasing use of computers as a communication tools, we can teach educational concepts in a charming and diverse atmosphere with the help of multimedia (25, 26).

Considering the importance of nutritional behaviour in preventing CRC, this study aimed to determine the effect of education, using multimedia based on HBM, on nutritional behaviour for CRC prevention.

## Materials and Methods

### Study Design

This clinical trial study was performed from November 2017 to March 2018 to evaluate the effects of HBM-based education on nutritional behaviours of CRC prevention in people over 50 years-old covered by three health centres in Bushehr, a city in the southwestern province of the same name in Iran.

The inclusion criteria of this study included being older than 50 years, having no history of CRC in their family, having a compact disc (CD) player, being able to read and write, and having a willingness to participate in the study, and exclusion criteria included inability to continue participation for at least 3 months.

The sample size was estimated at 45 subjects for each group based on a similar study (16) with using the Cochran formula (27) as $n_{1}=n_{2}=\frac{\left(z_{1-\frac{\alpha }{2}}+z_{1-\beta }\right)\left(S_{1}^{2}+S_{2}^{2}\right)}{{\left(\mu_{1}-\mu_{2}\right)}^{2}}$ with 1 – *β* = 90% power test and *α* set at 0.05 (two-tailed). In this formula, *μ* and *S* represent mean and standard deviation of total energy (kcal) in post-treatment between the HBM group and the control group, respectively, as in Abood’s study (16). The sample of 50 allowed attrition and detection of possibly smaller effects.

Among the 10 health centres, three centres were randomly selected; the names of the 10 centres were written on separate sheets of paper and three were randomly selected. After reviewing 6,807 records of people over 50 years-old in these centres, researcher determined the eligible individuals. Then, 120 participants were selected using Microsoft Excel software. After making phone calls to potential participants until 100 people willing to participate in the study were identified, information sheets for the participants at each centre were provided. These 100 people were randomly allocated into two groups (50 participants in each group); thus, people were divided into two groups based on the order in which they answered their telephones, so that the first person was assigned to the HBM group, the second to the control group, and so on.

**Figure 1:**
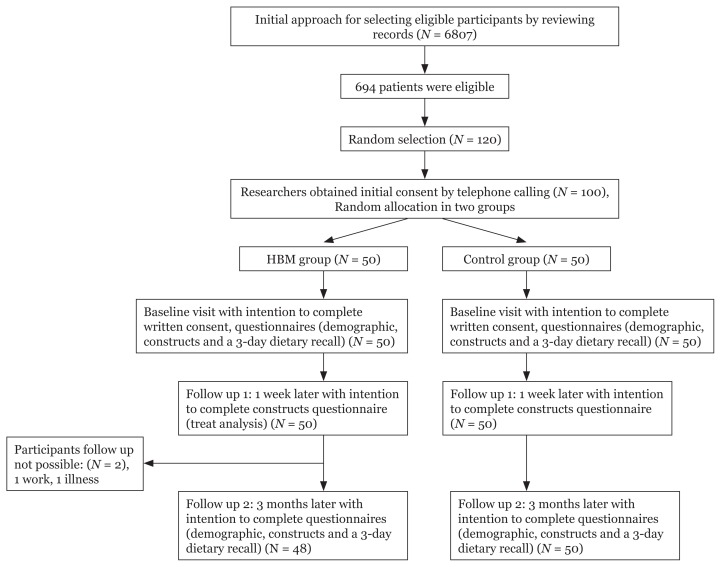
Consort flow chart of participants

From the 50 participants in the HBM group, two people were not willing to continue participation in the study and were excluded. Ultimately, 48 people in HBM group and 50 people in the control group completed the study (see Figure 1).

### Methods and Data Collection

At the beginning of the study, participants in both groups completed a written consent form and questionnaires, which collected demographic information, knowledge, and HBM constructs, and a three-day dietary recall. Completion of the questionnaire required 30 to 45 min. Then, the HBM group members received an audiovisual CD that contained 45 min of material. The following topics were covered by this educational CD:

1. Information about CRC; the prevalence and incidence of this cancer; and points about risk factors, including unhealthy dietary habits, such as a shift to a low-fibre diet with high fat and the consumption of ready meals, and their effective roles in the development of CRC (perceived susceptibility).
2. Tips about complications and problems associated with CRC and the high cost of treatment (perceived severity).
3. Information about the benefits of increasing the consumption of fruits and vegetables, the benefits of a high-fibre diet, the benefits of restricting meat and high-fat foods, the need to receive adequate levels of vitamin D, and the benefits of consuming foods containing folate and calcium for the prevention of CRC (perceived benefits).
4. Providing educational videos on how to cook a variety of healthy, non-meat and vegetable-based foods (increasing self-efficacy through performance accomplishment as well as reducing perceived barriers).

Follow-up on nutritional behaviour was done every 2 weeks in the first month and once in the second month by telephone, and people who changed their diet received verbal persuasion (based on their ability to recall the tips and increase self-efficacy through verbal persuasion). The educational CD was auto-run and was displayed by clicking on any field. A week after package delivery, researchers contacted each person to ensure that the educational CD had been opened by asking questions about its content. The participants of the control group did not receive training materials until the end of the study. Participants in both groups, 1 week after the completion of the first phase of questionnaires, completed the questionnaires related to knowledge and HBM constructs and, 3 months after the second stage, completed questionnaires related to knowledge, HBM constructs, and a three-day dietary recall.

### Instruments and Measures

The final questionnaire included three sections comprised of demographic factors, questions regarding the patient’s knowledge and HBM constructs, and a three-day dietary recall questionnaire. The first section of the questionnaire evaluated demographics and predisposing factors for CRC, including history of constipation (disposing of dry stool less than three times a week), gastrointestinal disorders, physical activity (one item that evaluated a self-report of physical activity duration in the last week with three levels—no activity, less than 150 min, and 150 min or more per week), and use of tobacco (use of cigar or water pipes in the past month).

The section related to knowledge and HBM constructs included 64 items. Knowledge regarding CRC was evaluated by 10 items, which included right answers (1 point) and wrong answers (0 points). The HBM construct questionnaire include perceived susceptibility (six items), perceived severity (15 items), perceived self-efficacy (10 items), perceived benefits (nine items), and perceived barriers (14 items). Items related to perceived susceptibility, and severity constructs were extracted from Tahmasebi et al.’s study (28); items of other constructs were self-developed. All items in the constructs were scored using a Likert scale ranging from 1 (strongly disagree) to 5 (strongly agree) with a higher ranking on the Likert scale indicating greater agreement with the health beliefs that were assessed.

The third section of the questionnaire measured the nutritional behaviour with a three-day dietary recall questionnaire. The questionnaire was completed through a semi-structured interview. In the interview, subjects were asked to recall their diet diaries over the last 3 days, which included two working days and one holiday. In this study, dietary micronutrients intake was calculated by Nutritionist IV software; additionally, the number of food servings consumed, the intake of dietary micronutrients (such as folate, calcium, vitamin D), and saturated fatty acids were evaluated.

Ten experts, including eight health education specialists and two nurses whose research area was CRC, were asked to evaluate content validity of the constructs using the content validity ratio (CVR). For this purpose, the panel assessed each item using a three-point Likert-type scale, where 1 = essential, 2 = useful but not essential, and 3 = unessential. For the 10-expert panel, a CVR score of 0.62 or higher indicates good content validity (29). The CVR score for constructs was calculated at 0.71 (perceived severity) to 0.91 (perceived benefits) and was acceptable. The reliability of constructs according to Cronbach’s alpha coefficients ranged from 0.73 (perceived susceptibility and severity) to 0.88 (perceived self-efficacy).

The data was analysed by the Statistical Package for the Social Sciences software, version 22.0. Descriptive statistics, a Chi-square test, independent and paired *t*-test, repeated measurement ANOVA, a Wilcoxon signed-rank test, and a Mann-Whitney test were used for data analysis. The Chi-square test was used to compare the two groups with respect to the qualitative variables, and an independent *t*-test was utilised for quantitative variables such as age. Paired sample *t*-test was used to compare knowledge and constructs between two time points in each group. The repeated measurement ANOVA was used to compare the mean scores of constructs within and between the groups. The Mann-Whitney test was used to evaluate the mean difference of food servings and dietary micronutrients intake before (baseline) and 3 months later (second follow-up) in the two groups, and a Wilcoxon signed-rank test was used to evaluate the mean difference of food servings and dietary micronutrients intake at the baseline and three month later in each group because mean differences did not have normal distribution. A *P*-value of less than 0.05 was considered statistically significant.

### Ethical Considerations

To observe ethics in the research, participants could be excluded from the study at any time, and all the information collected was confidential and anonymous. Written informed consent was received from all participants.

## Results

In total, 48 participants in the HBM group and 50 participants in the control group completed this study. At the baseline test, there were no significant differences between the HBM and control groups regarding demographic and predisposing factors. For instance, the average age of participants in the HBM group was 55.60 (4.17), and the average age in the control group was 57.74 (6.43) (*P* = 0.055). The mean (SD) BMI in the HBM group and control group was 26.77 (3.95) and 27.46 (4.17), respectively (*P* = 0.404). Also, in terms of predisposing factors for CRC, the two groups were similar. For instance, in the HBM group 20.8% (*n* = 10) of participants and in the control group 12% (*n* = 6) smoked (*P* = 0.282). Other demographic characteristics and predisposing factors of CRC are shown in Table 1.

Also, the participants in the HBM and the control groups were similar in their health beliefs related to CRC and nutritional behaviour of CRC prevention at baseline. However, the differences between the two groups were statistically significant after education in several constructs (refer to Table 2). Comparison of pre- and post-test results (1 week and 3 months later) in the HBM group by repeated measures ANOVA found that changes occurred in all of the constructs except perceived susceptibility (*P* = 0.356) and perceived barriers (*P* = 0.118). The results demonstrated an increase in knowledge, perceived severity, perceived self-efficacy, and perceived benefits. Based on the paired *t*-test, there were significant changes in baseline and 1 week later, baseline and 3 months later, and 1 week and 3 months later in knowledge, severity, and benefit (*P* < 0.001). Also, there were significant changes in baseline and 3 months later, and 1 week and 3 months later in self-efficacy (*P* < 0.001).

However, in the control group, the results showed that there were no significant changes between the pre- and post-test scores for all constructs. Comparison of the two groups during that time showed no notable differences in the scores of perceived susceptibility (*P* = 0.746) and perceived barriers (*P* = 0.099), but knowledge, perceived severity, self-efficacy, and benefits were perceived to be higher in the HBM group compared to the control group (see Table 2).

Based on the Wilcoxon signed-rank test, the results demonstrated a significant increase in fruit and vegetable consumption (*P* < 0.001) and dairy intake (*P* = 0.001) in the HBM group after education. Also, there was a significant increase in micronutrients, such as vitamin D (*P* = 0.003), folate (*P* = 0.003), calcium (*P* = 0.026), and dietary fibre (*P* = 0.011) in the HBM group. In the control group, the results demonstrated a significant increase in bread serving intake (*P* = 0.006) and red meat (*P* = 0.045), and significant decreases in fruit and vegetable (*P* = 0.002) and white meat servings (*P* = 0.011) after education. Also, there was a significant decrease in vitamin D (*P* = 0.004), folate (*P* < 0.001), and dietary fibre (*P* < 0.001) in the control group after education (see Table 3).

Comparison of the mean differences of food servings and dietary micronutrient intake before and 3 months after education in both groups showed that the mean difference in fruit and vegetable (*P* < 0.001) and dairy servings (*P* = 0.001) as well as vitamin D (*P* < 0.001), folate (*P* < 0.001), calcium (*P* = 0.008), and dietary fibre intake (*P* < 0.001) increased, while red meat servings (*P* = 0.016) decreased in the HBM group compared to the control group (see Table 4).

## Discussion

The goal of this study was to evaluate an HBM-based nutrition intervention for CRC prevention in people over 50 years-old in Iran. The intervention was designed to change dietary behaviour in a positive manner by modifying specific health beliefs while increasing nutrition knowledge and improving dietary behaviours related to CRC through multimedia education. Health beliefs, except perceived susceptibility and perceived barriers, significantly changed.

Intervention with the use of the educational CD was successful and appears to be associated with producing significant increases in nutrition knowledge and significantly decreasing red meat consumption while increasing fruit and vegetable, and dairy intake. In this study, micronutrients, such as vitamin D, folate, calcium, and dietary fibre, increased, but education failed to reduce energy intake and consumption of saturated fat. This supports our hypothesis that a health education program based on HBM can be effective in promoting the adoption of nutritional behaviours to prevent CRC.

Increasing the participants’ knowledge of the need for CRC prevention is likely to improve the participants’ healthy behaviour (23). Participants’ knowledge of CRC significantly increased after multimedia education in the HBM group. The increase in awareness in this study is also consistent with the findings of several other studies (16, 30–34). Research indicates that dietary interventions grounded in behaviour change theory while changing health beliefs are effective in changing diet (5). Our findings showed that perceived severity, self-efficacy, and benefits regarding nutritional behaviour of CRC prevention were significantly increased after multimedia education in the HBM group, which are consistent with the findings of several other studies (16, 35, 36).

Among the constructs of the health belief model, the perceived susceptibility and barriers were not changed after education. Previous studies of the relationships between healthful behaviours and HBM constructs have also noted that perceived susceptibility and healthful behaviours are not always related (18, 37), and in Wallace’s study, a small effect was observed for perceived susceptibility of osteoporosis (38). These findings are compatible with the findings of the current study.

An extensive review of the literature has shown that the strongest predictor of nutrition-related behaviour change is the benefit-cost ratio, so that when a concrete change in nutrition behaviour occurs, the perceived benefits of the behaviour outweighs the perceived barriers (16, 37, 39). In spite of the fact that this study showed that no significant change was made in perceived barriers, increased perceived benefits caused a change in nutritional behaviour, which is congruent with previous studies.

In this study, multimedia education based on HBM increased the consumption of fruits and vegetables and dairy products and increased the intake of micronutrients, including vitamin D, folate, calcium, and dietary fibre. In Manios’s study, education based on HBM increased consumption of dairy products and intake of calcium and vitamin D (32). Also, in Alidosti’s study, changes in health beliefs led to an increase in the consumption of fruits and vegetables and dairy products (40).

In Abood’s study, an increase in the perceived benefits of a healthy diet reduced fat intake but was not effective in changing the consumption of fruits and vegetables (16), which contradicts the results of the present study. In the current study, education failed to reduce saturated fat intake because the focus of the intervention was on increasing fruit and vegetable intake, with the anticipation that behaviour maintenance or self-change would spontaneously occur in regard to a low-fat diet. The positive change in increasing fruit and vegetable intake is consistent with the focus and emphasis of the intervention and instruction. Also, in previous studies, the participants reported more barriers for decreasing fat intake than increasing fruit and vegetable intake (41); additionally, adding foods to a diet is probably more comfortable than restricting food intake. Thus, adding fruits or vegetables may have been easier than lowering total fat intake.

The present study may have limitations. Because diet was not observed, self-report bias may have occurred. Nevertheless, self-report, because of its practicality and realism, is the norm in nutrition studies. Also, some participants delayed their use of the educational CD, citing having too much work to do as the reason why. Also, we do not claim that completely random selection was performed, but random allocation into the two groups was done.

## Conclusion

According to the results obtained, it seems that design and implementation of a training program using HBM has positive impacts on the changes of dietary behaviour for CRC prevention, and the use of multimedia, due to its charm and ease of use, seems to be a desirable medium for changing nutritional behaviour.

## Figures and Tables

**Table 1:** Comparison of demographic and predisposing factors between HBM (*n* = 48) and control (*n* = 50) groups among people over 50 years old

Variables			HBM group		Control group		Statistics	*P*-value
	
			*n*	%	*n*	%		
Demographic factors	Gender	Male	26	54.2	26	52.0	0.046	0.830
		Female	22	45.8	24	48.0		
	Education level	Less than Diploma	21	43.8	24	48.0	0.178	0.673
		Diploma or higher	27	56.2	26	52.0		
	Job	Housekeeper	18	37.5	23	46.0	1.592	0.661
		Employee	3	6.3	5	10.0		
		Pensionary	18	37.5	15	30.0		
		Others	9	19.7	7	14.0		
	Married status	Married	44	91.7	42	84.0	1.340	0.247
		Single or divorce	4	8.3	8	16.0		

Predisposing factors	History of constipation in last month	Yes	14	29.2	17	34.0	0.265	0.607
		No	34	70.8	33	66.0		
	Refer to doctor for gastric disorders	Yes	16	33.3	18	36.0	0.077	0.782
		No	32	66.7	32	64.0		
	Physical activity	No activity	18	37.5	26	52.0	2.115	0.347
		Under 150 min/week	13	27.1	11	22.0		
		Upper 150 min/week	17	35.4	13	26.0		
	Smoking	Yes	10	20.8	6	12.0	1.399	0.237
		No	38	79.2	44	88.0		

**Table 2:** Constructs’ scores of health belief model in people over 50 years in HBM (*n* = 48) and control group (*n* = 50) in during the study

Constructs	HBM group mean (SD)			Control group mean (SD)			*P*-value^a^
	
	Baseline	1 week	3 months	Baseline	1 week	3 months	
Susceptibility	2.88 (0.42)	2.80 (0.44)	2.81 (0.44)	2.86 (0.45)	2.75 (0.43)	2.72 (0.47)	0.746
Severity	3.89 (0.48)	4.43 (0.35)	4.55 (0.31)*	3.99 (0.44)	4.02 (0.40)	3.98 (0.36)	< 0.001
Benefits	4.19 (0.35)	4.48 (0.30)	4.73 (0.22)*	4.03 (0.38)	4.05 (0.37)	4.00 (0.38)	< 0.001
Barrier	2.49 (0.48)	2.38 (0.48)	2.35 (0.49)	2.57 (0.45)	2.57 (0.46)	2.61 (0.48)	0.099
Self-efficacy	3.81 (0.65)	3.88 (0.53)	4.31 (0.53)*	3.54 (0.75)	3.50 (0.64)	3.38 (0.71)	< 0.001
Knowledge	0.59 (0.19)	0.81 (0.09)	0.85 (0.09)*	0.52 (0.19)	0.57 (0.20)	0.56 (0.20)	< 0.001

a Comparison of mean change in during time between two groups using repeated measurement ANOVA

* The changes in mean score during the time in each groups using repeated measurement ANOVA (*P* < 0.001)

**Table 3:** Comparison of food serving and micronutrients intake during baseline and 3 months later in HBM (*n* = 48) and control group (*n* = 50) in people over 50 years

Food & micronutrients type		HBM group Median (IQR)		*P*-value*	Control group Median (IQR)		*P*-value*
	
		Baseline	3 months		Baseline	3 months	
Food serving intake	Bread & cereal	8.83 (3.79)	9.67 (3.25)	0.085	8.33 (4.25)	9.25 (4.33)	0.006
	Fruit & vegetable	2.50 (2.13)	4.83 (3.33)	< 0.001	3.00 (1.88)	2.58 (1.38)	< 0.002
	Dairy	0.33 (0.67)	0.67 (1.00)	0.001	0.33 (0.54)	0.33 (0.67)	0.976
	Red meat	0.17 (0.50)	0.33 (0.33)	0.313	0.33 (0.67)	0.67 (0.67)	0.045
	White meat	0.67 (0.63)	0.50 (0.33)	0.063	0.67 (0.67)	0.50 (0.33)	0.011

Micronutrient intake	Vitamin D (mcg)	0.12 (0.84)	1.24 (3.30)	0.003	0.62 (1.24)	0.20 (0.62)	0.004
	Folate (mcg)	368.20 (360.30)	536.45 (356.98)	0.003	386.05 (296.03)	263.35 (236.55)	< 0.001
	Calcium (mg)	1125.00 (967.03)	1396.50 (995.75)	0.026	1176.00 (806.73)	1055.50 (751.47)	0.059
	Saturated fatty acid(g)	17.37 (11.45)	20.35 (15.40)	0.553	15.79 (16.28)	18.06 (14.56)	0.612
	Dietary fiber (g)	24.26 (20.88)	35.61 (21.49)	0.011	27.18 (15.30)	19.52 (14.83)	< 0.001
	Energy intake (kcal)	3287.00 (1551.00)	3721.00 (1644.25)	0.924	3439.50 (2202.50)	3043.00 (1591.50)	0.149

* Using Wilcoxon signed ranks test

**Table 4:** Comparison of mean changes of food serving and micronutrients intake between HBM (*n* = 48) and control group (*n* = 50) in people over 50 years

Food & micronutrients type		HBM group Median (IQR)	Control group Median (IQR)	*P*-value *
Food serving intake	Bread & cereal	0.67 (3.42)	0.50 (2.16)	0.809
	Fruit & vegetable	2.00 (2.17)	−0.58 (1.17)	< 0.001
	Dairy	0.33 (1.00)	0.00 (0.50)	0.001
	Red meat	0.00 (0.50)	0.33 (0.67)	0.016
	White meat	−0.08 (0.50)	−0.33 (0.33)	0.539

Micronutrient intake	Vitamin D (mcg)	0.62 (3.01)	0.00 (0.62)	< 0.001
	Folate (mcg)	110.75 (437.15)	−150.60 (234.90)	< 0.001
	Calcium (mg)	343.50 (1326.80)	−164.50 (237.80)	0.008
	Saturated fatty acid (g)	0.58 (18.57)	−1.64 (9.27)	0.439
	Dietary fiber (g)	4.35 (23.30)	−4.83 (13.02)	< 0.001
	Energy intake (kcal)	−133.00 (2031.00)	−354.00 (1167.00)	0.519

* Using Mann-Whitney Test

## References

[1] Williams CD, Satia JA, Adair LS, Stevens J, Galanko J, Keku TO (2009). Dietary patterns, food groups, and rectal cancer risk in Whites and African-Americans. Cancer Epidemiology and Prevention Biomarkers.

[2] Dolatkhah R, Somi MH, Asvadi Kermani I, Ghojazadeh M, Asghari Jafarabadi M, Farassati F (2015). Increased colorectal cancer incidence in Iran: a systematic review and meta-analysis. BMC Public Health.

[3] Marley AR, Nan H (2016). Epidemiology of colorectal cancer. Int J Mol Epidemiol Genet.

[4] Vahdatimanesh Z, Zendehdel K, Sari AA, Farhan F, Nahvijou A, Delavari A (2017). Economic burden of colorectal cancer in Iran in 2012. Med J Islam Repub Iran.

[5] Avery KNL, Donovan JL, Horwood J, Lane JA (2013). Behavior theory for dietary interventions for cancer prevention: a systematic review of utilization and effectiveness in creating behavior change. Cancer Causes Control.

[6] Rafiemanesh H, Pakzad R, Abedi M, Kor Y, Moludi J, Towhidi F (2016). Colorectal cancer in Iran: epidemiology and morphology trends. EXCLI J.

[7] Bishehsari F, Mahdavinia M, Vacca M, Malekzadeh R, Mariani-Costantini R (2014). Epidemiological transition of colorectal cancer in developing countries: environmental factors, molecular pathways, and opportunities for prevention. World J Gastroenterol.

[8] Harris R (2016). Global epidemiology of cancer.

[9] Azizi F, Allahverdian S, Mirmiran P, Rahmani M, Mohammadi F (2001). Dietary factors and body mass index in a group of Iranian adolescents: Tehran lipid and glucose study-2. Int J Vitam Nutr Res.

[10] Ghassemi H, Harrison G, Mohammad K (2002). An accelerated nutrition transition in Iran. Publ Health Nutr.

[11] Hosseini SV, Izadpanah A, Yarmohammadi H (2004). Epidemiological changes in colorectal cancer in Shiraz, Iran: 1980–2000. ANZ J Surg.

[12] Duthie SJ (1999). Folic acid deficiency and cancer: mechanisms of DNA instability. Br Med Bull.

[13] Gorham ED, Garland CF, Garland FC, Grant WB, Mohr SB, Lipkin M (2005). Vitamin D and prevention of colorectal cancer. J Steroid Biochem Mol Biol.

[14] Schmid D, Leitzmann M (2014). Association between physical activity and mortality among breast cancer and colorectal cancer survivors: a systematic review and meta-analysis. Ann Oncol.

[15] Baena R, Salinas P (2015). Diet and colorectal cancer. Maturitas.

[16] Abood DA, Black DR, Feral D (2003). Nutrition education worksite intervention for university staff: application of the health belief model. J Nutr Educ Behav.

[17] Deshipande S, Basil MD, Bas DZ (2009). Factors influencing healthy eating habits among college students: an application of the health belief model. Health Mark Q.

[18] Hanson JA, Benedict JA (2002). Use of the health belief model to examine older adults’ food-handling behaviors. J Nutr Educ Behav.

[19] Kim HS, Ahn J, No JK (2012). Applying the Health Belief Model to college students’ health behavior. Nutr Res Pract.

[20] Sahraee A, Noroozi A, Tahmasebi R (2013). Predicting factors of breast self-examination based on health belief model and locus of control among women aged 20–50 years. J of Hayat.

[21] Lori WT, Sharon BH, DiBrezzo R, Ches J (2004). Design and implementation of an osteoporosis prevention program using the health. American Journal of Health Studies.

[22] Hazavehei SM, Taghdisi MH, Saidi M (2007). Application of the health belief model for osteoporosis prevention among middle school girl students, Garmsar, Iran. Educ Health.

[23] Ghaffari M, Tavassoli E, Esmaillzadeh A, Hassanzadeh A (2012). Effect of Health Belief Model based intervention on promoting nutritional behaviors about osteoporosis prevention among students of female middle schools in Isfahan, Iran. J Edu Health Promot.

[24] Sharifirad G, Entezari MH, Kamran A, Azadbakht L (2009). The effectiveness of nutritional education on the knowledge of diabetic patients using the health belief model. J Res Med Sci.

[25] Khan MA, Shah S, Grudzien A, Onyejekwe N, Banskota P, Karim S (2011). A diabetes education multimedia program in the waiting room setting. Diabetes Ther.

[26] Bell AM, Fonda SJ, Walker MS, Schmidt V, Vigersky RA (2012). Mobile phone-based video messages for diabetes self-care support. J Diabetes Sci Technol.

[27] Cochran WG (1963). Sampling techniques.

[28] Tahmasebi R, Noroozi A, Dashdebi KG (2016). Psychometric evaluation of the colorectal cancer screening belief scale based on Health Belief Model’s constructs for the fecal occult blood test. Asian Pac J Cancer Prev.

[29] Lawshe CH (1975). A quantitative approach to content validity. Pers Psychol.

[30] Lin W, Yang H-C, Hang C-M, Pan W-H (2007). Nutrition knowledge, attitude, and behavior of Taiwanese elementary school children. Asia Pac J Clin Nutr.

[31] Anderson A, Porteous L, Foster E, Higgins C, Stead M, Hetherington M (2005). The impact of a school-based nutrition education intervention on dietary intake and cognitive and attitudinal variables relating to fruits and vegetables. Public Health Nutr.

[32] Manios Y, Moschonis G, Katsaroli I, Grammatikaki E, Tanagra S (2007). Changes in diet quality score, macro-and micronutrients intake following a nutrition education intervention in postmenopausal women. J Hum Nutr Diet.

[33] Gammage KL, Francoeur C, Mack DE, Klentrou P (2009). Osteoporosis health beliefs and knowledge in college students: the role of dietary restraint. Eat Behav.

[34] Park S, Chang S, Chung C (2005). Effects of a cognition-emotion focused program to increase public participation in Papanicolaou smear screening. Public Health Nurs.

[35] Sol BG, van der Graaf Y, van Petersen R, Visseren FL (2011). The effect of self-efficacy on cardiovascular lifestyle. Eur J Cardiovasc Nurs.

[36] Ashford S, Edmunds J, French DP (2010). What is the best way to change self-efficacy to promote lifestyle and recreational physical activity? A systematic review with meta-analysis. Br J Health Psychol.

[37] Vassallo M, Saba A, Arvola A, Dean M, Messina F, Winkelmann M (2009). Willingness to use functional breads: applying the health belief model across four European countries. Appetite.

[38] Wallace LS (2002). Osteoporosis prevention in college women: application of the expanded health belief model. Am J Health Behav.

[39] Lotfi Mainbolagh B, Rakhshani F, Zareban I, Montazerifar F, Alizadeh Sivaki H, Parvizi Z (2012). The effect of peer education based on health belief model on nutrition behaviors in primary school boys. J Research Health.

[40] Alidosti M, Sharifirad G, Hemate Z, Delaram M, Najimi A, Tavassoli E (2011). The effect of education based on health belief model of nutritional behaviors associated with gastric cancer in housewives of Isfahan city. Sci Res J Shahed Univ.

[41] Contento IR, Murphy BM (1990). Psycho-social factors differentiating people who reported making desirable changes in their diets from those who did not. J Nutr Educ Behav.

